# Internal Stress Monitoring of In-Service Structural Steel Members with Ultrasonic Method

**DOI:** 10.3390/ma9040223

**Published:** 2016-03-23

**Authors:** Zuohua Li, Jingbo He, Jun Teng, Ying Wang

**Affiliations:** 1 School of Civil and Environment Engineering, Shenzhen Graduate School, Harbin Institute of Technology, Shenzhen 518055, China; lizuohua@hit.edu.cn (Z.L.); hejingbo@stmail.hitsz.edu.cn (J.H.); 2 IoT Application Technology Center of NDT, Shenzhen Graduate School, Harbin Institute of Technology, Shenzhen 518055, China; 3 Department of Civil and Environmental Engineering, University of Surrey, Guildford GU2 7XH, UK

**Keywords:** ultrasonic method, longitudinal critically refracted waves, in-service structural steel members, internal stress, acoustoelasticity

## Abstract

Internal stress in structural steel members is an important parameter for steel structures in their design, construction, and service stages. However, it is hard to measure via traditional approaches. Among the existing non-destructive testing (NDT) methods, the ultrasonic method has received the most research attention. Longitudinal critically refracted (Lcr) waves, which propagate parallel to the surface of the material within an effective depth, have shown great potential as an effective stress measurement approach. This paper presents a systematic non-destructive evaluation method to determine the internal stress in in-service structural steel members using Lcr waves. Based on theory of acoustoelasticity, a stress evaluation formula is derived. Factor of stress to acoustic time difference is used to describe the relationship between stress and measurable acoustic results. A testing facility is developed and used to demonstrate the performance of the proposed method. Two steel members are measured by using the proposed method and the traditional strain gauge method for verification. Parametric studies are performed on three steel members and the aluminum plate to investigate the factors that influence the testing results. The results show that the proposed method is effective and accurate for determining stress in in-service structural steel members.

## 1. Introduction

### 1.1. Internal Stress and Stress Measurement Methods

Internal stress in in-service structural steel members plays an important role in the design and analysis of steel structures. The effective and reliable measurement of internal stress can provide useful information that allows the safety of existing structures to be evaluated. For example, stress concentration could lead to abnormal functioning and even structural failures. The early detection of problematic stress concentrations may help asset managers to solve the issue in advance, which can minimize the risk of structural malfunction and failure. However, in current practices, the stresses in in-service structural members are usually calculated indirectly based on the design draft and the measured or estimated loading, because accurate and reliable measurement of in-service stresses through traditional approaches is difficult. This results in the differences between estimated and actual stress conditions, which may increase the risk of structural failure.

Traditional stress measurement methods include the sectioning method, hole-drilling method, strain gauge method, X-ray diffraction, magnetic-elastic method, and neutron diffraction method. The sectioning method [1] and hole-drilling method [2] are both destructive to members and thus are not suggested to be used in existing structures. The strain gauge method [3] can be used to determine stress values indirectly by measuring the strain change on the surface of the structural member. However, it can only measure stress change, instead of the absolute value of stress. In addition, only stress at the structural surface can be measured by using strain gauges. The X-ray diffraction method [4] can determine the surface strain by measuring intercrystalline distance of a crystal structure according to Bragg’s law. It is non-destructive, but only allows measurement of stresses to a depth of 10 µm. Neutron diffraction method [5] is very similar to the X-ray method as it relies on elastic deformations within a polycrystalline material that cause changes in the spacing of the lattice planes from their stress-free condition. The equipment for this method is complex and therefore not suitable for field testing. The magnetic elastic method [6] is based on the Barkhausen effect; it is limited by magnetization conditions and the complex equipment required. Its measurement reliability and precision are inferior to those of other methods. Therefore, the traditional methods described above are unsuitable for efficiently measuring internal stress in steel structure members.

In recent years, ultrasonic methods using piezoelectric transducers have been proposed to detect and identify damage in complex structures [7,8]. However, studies that investigate the application of ultrasonic methods to measure the internal stress levels of in-service structural steel members remain rare in the literature.

### 1.2. Ultrasonic Stress Measurement

Ultrasonic wave propagation in a solid medium is affected by the internal stress of the medium. This property makes non-destructive measurement of internal stress possible. A linear relationship can be found between ultrasonic velocity and material stress, which is called acoustoelasticity. In the range of elasticity, the time-of-flight (TOF) of ultrasonic waves presents a linear relationship with material stress when a wave propagates in a fixed acoustic path. According to finite deformation theory, Hughes [9] proposed a formula for isotropic materials based on acoustoelasticity theory. Based on this, a stress measurement method was first proposed in 1950. Crecraft [10] formulated the stress-induced velocity variations of both longitudinal and shear waves for steel, aluminum, and copper through experiments; the corresponding third-order elastic constants were also obtained. Makhort [11] described the theory of the propagation of longitudinal, shear, and surface Rayleigh waves in initially stressed members, and proposed the basic approaches and principles of the ultrasonic non-destructive technique for finding biaxial and triaxial stresses in solids. On this basis, an internal stress formula was derived, and the method was verified through a test on polymeric material. Compared with traditional methods, ultrasonic stress measurement methods have advantages in terms of both cost and flexibility; and are regarded as a promising approach for the non-destructive evaluation of stress [12].

Internal stress measurement using ultrasonic methods can be classified into three groups according to the different wave types employed. The first group aims to directly use TOF, mainly for longitudinal waves. Joshi [13] used longitudinal waves to measure the axial stress in bolts. Because the variation of ultrasonic wave velocity in the range of actual stress in the bolt is very small, the determination of stress requires precise and accurate measurement of the wave velocity. The phase detection method [14] was then used for the precise measurement of TOF; and the experimental results showed that ultrasonic wave velocity decreases linearly with the increase of stress. Chaki [15] used the velocity ratio (the difference in the acoustoelastic coefficients of longitudinal waves and transverse waves) method to measure the axial stress in bolts. This method is more effective because the value of axial load is calculated from the ratio of TOF in the stressed state only, without accounting for the TOF measurement in the unstressed state. The TOF variation and frequency shift of longitudinal waves are very small, and thus the stress-induced velocity changes are very difficult to detect. For this reason, a data acquisition device with a high sampling rate is needed and this increases the cost of this approach. A number of factors, including the material-microstructure effects, environmental noise, and the bonding layer thickness, can negatively affect the measurement accuracy of the TOF method. These factors make *in situ* measurement difficult.

The second group combines information from both longitudinal and transverse waves for stress measurement. In the railroad industry, temperature changes lead to stresses in segments of seamless railway track. In addition, performing stress measurements on active railway routes is a dangerous task. To overcome these challenges, Szelazek [16] used a birefringence based approach to measure the stress on the train wheel rim caused by braking. This method determines the stress of the wheel rim by measuring the TOFs of both longitudinal and shear waves propagating perpendicularly to the acoustic stress field. The stress measured by this method is the mean value of the whole rim and cannot reflect the gradient distribution of the stress in the whole rim. Yasui and Kawashima [17] used a broadband normal incidence transducer, which excited and received a 10 MHz longitudinal wave and a 5 MHz transverse wave simultaneously, to measure the axial stress of a bolt. Unfortunately, employing both longitudinal and transverse waves to measure stress is complex and time-consuming. Therefore, it is not a feasible method in practice.

The third group employs critically refracted longitudinal (Lcr) waves to measure stress. Since the variation of Lcr wave velocity due to stress change is much greater than those of other types of waves, they become the best candidates to be used for the stress evaluation [18]. The most common application of this approach is for residual stress measurement after welding. Welding residual stress results from complex thermo-plastic deformations in the material [19,20,21]. The sensitivity of ultrasonic waves to stress has been tested by Egle and Bray [18] with a pearlitic steel bar under tensile and compressive loads. Among all ultrasonic waves, Lcr waves exhibited the largest sensitivity to stress. Therefore, the Lcr wave method has been the focus of many studies recently. Bray and Santos [19,20] used Lcr waves to detect the residual stresses of an aluminum welded seam and a steel welded seam. The results confirmed the relaxation phenomenon of welding residual stress, and that the stress relaxation of the aluminum welded seam was less than that of the steel counterpart. Javadi [21,22,23,24] used Lcr waves to measure the overall distribution of welding residual stress in an austenitic stainless steel member using friction stir welding. Experimental results were verified by the hole-drilling method and theoretical analysis. Based on this, welding residual stress distribution at different depths was obtained. Combining this with numerical simulation results using ANSYS, the diagram of stress distribution was drawn. The majority of studies on Lcr wave are still in their early stages, with a limited number of industrial applications. There are three possible reasons. First, ultrasonic wave propagation characteristics are influenced by multiple factors including grain size [25], carbon content [26], material texture [27], temperature [28], water environment [29,30], and coupling conditions [31]. All these factors present different challenges and can increase the difficulty level of accurate stress measurement. Second, stress measured by this approach represents the average value in a fixed path and may result in noticeable errors in residual stress sections with high gradients. Third, when using the Lcr wave method, some parameters need to be measured on in-service structural members. For example, the elastic modulus of steel members needs to be measured by using the tensile test, and Lcr travel time data in in-service structural steel members under a “stress-free” condition need to be collected. These may not be practical in many applications. Internal stress of in-service structural steel members is one of the mechanical stress. It is present in materials with external loading and has an associated length change according to the loading form, which is different from residual stress. Compared to massive studies on the measurement of residue stresses, studies to measure the internal stress of in-service structural steel members remain rare in the literature. 

### 1.3. Goals and Objectives of This Study

Realizing the above difficulties, this paper aims to propose a practical non-destructive evaluation approach to determine the internal stress of in-service structural steel members using Lcr waves, and the ultimate objective of this study is for industrial application. In accordance with the characteristics of in-service structural steel members, a stress evaluation formula is derived. A comprehensive testing equipment, including both hardware and software systems, is designed and developed in the laboratory. Six structural members are tested using the proposed method. The results of two members are validated by using the traditional strain gauge method. The influencing factors, such as the position of probes, materials, and ultrasonic path, are investigated by combining theoretical and experimental studies on the other four specimens.

## 2. Theory

Hughes and Kelly [32] derived the relationship between elastic wave velocity and stress for the theory of acoustoelasticity. Tokuoka and Iwashimizu [33] further developed the theory based on the following four basic assumptions: (1) continuity for a solid body; (2) small perturbation of ultrasonic waves are superimposed on the finite static deformation of the object; (3) the deformations of objects are super elastic and uniform; (4) the object deformation process is isentropic. Based on these studies, when the ultrasonic longitudinal wave reaches an interface between two media with different acoustic impedance, wave mode conversion occurs. At that time, one part of the energy is reflected by the interface back to the first medium, and the reflected angle equals to the incident angle. The other part of the energy refracts into the second medium, and the refracted longitudinal wave and shear wave are generated. According to Snell’s law [34], when ultrasonic velocity in the second medium is greater than in the first one, the angle between the refracted longitudinal wave and shear wave increases with the increase of the incidence angle of the longitudinal wave. When the incident angle increases to a certain value (different for different materials), the refracted longitudinal wave travels parallel to member surface. Now, the refracted longitudinal wave becomes the critically refracted longitudinal wave, namely, the Lcr wave. As a special ultrasonic wave mode, the Lcr wave travels parallel to the member surface and propagates beneath the surface at a certain depth. The propagation depth of the Lcr wave is a function of frequency, but an explicit form of this function has not yet been derived [22]. 

In this study, structural steel member material is assumed to be isotropic and homogeneous. Further, it is assumed to be in its elastic range. The speed of the Lcr wave traveling parallel to the load can be related to stress by the following expression [35]:

(1) $$\rho_{0}V^{2}=\lambda +2\mu +\frac{\sigma }{3\lambda +2\mu }\left[\frac{\lambda +\mu }{\mu }\left(4\lambda +10\mu +4m\right)+\lambda +2l\right]$$

where ρ_0_ is the material density before deformation; V is the velocity of the Lcr wave when the steel member is under stress; σ is the stress of the steel member; λ and μ are the second order elastic constants of the material; and l, m, and n are the third order elastic constants of the material. Equation (1) illustrates that stress is a complex function of second order wave velocity, and it is influenced by the materials’ second and third order elastic constants. Therefore, the relationship between stress and velocity is not practically convenient for stress measurement. 

To allow feasible stress measurements, this paper proposed the following steps to simplify the relationship between stress and velocity.

When the steel member is under zero-stress condition, the velocity of the Lcr wave is:

(2) $$\rho_{0}{V_{0}}^{2}=\lambda +2\mu$$

where V_0_ is the Lcr wave velocity when steel member is under zero-stress condition. By substituting Equation (2) into Equation (1), a new formula can be obtained:

(3) $$V^{2}=V_{0}^{2}(1+k\sigma )$$

where k is a constant related to the material properties:

(4) $$k=\frac{\frac{4\lambda +10\mu +4m}{\mu }+\frac{2l-3\lambda -10\mu -4m}{\lambda +2\mu }}{3\lambda +2\mu }$$

The Equation (3) can be written as the following form:

(5) $$\sigma =\frac{1}{k}\cdot (\frac{V^{2}}{V_{0}^{2}}-1)$$

The second order Taylor series expansion of Equation (5) about the point V = V_0_ can be written as:

(6) $$\sigma =\frac{2}{k}\cdot \frac{V-V_{0}}{V_{0}}+\frac{3}{k}\cdot \frac{{\left(V-V_{0}\right)}^{2}}{V_{0}^{2}}$$

The velocity change of Lcr wave caused by stress is too small to be measured, so further simplification is necessary. Based on the fixed acoustic path method [36], this study transforms the relationship between stress and velocity to the relationship between stress and TOF in a fixed acoustic path, L.

The TOF of Lcr waves in the path are t_0_ and t when steel member is under zero-stress and stress conditions, respectively. By substituting $V=\frac{L}{t}$, and $V_{0}=\frac{L}{t_{0}}$, into Equation (6), it becomes:

(7) $$\sigma =\frac{1}{k}\cdot (\frac{3t_{0}^{2}}{t^{2}}-\frac{4t_{0}}{t}+1)$$

By expanding Equation (7) in the Taylor series about the point t = t_0_, the following formula can be obtained:

(8) $$\sigma =\frac{1}{k}\cdot [\frac{2}{t_{0}}\cdot (t_{0}-t)+\frac{5}{t_{0}^{2}}\cdot {\left(t_{0}-t\right)}^{2}+\frac{8}{t_{0}^{3}}\cdot {\left(t_{0}-t\right)}^{3}+\cdot \cdot \cdot \cdot \cdot \cdot +{\left(-1\right)}^{n}\cdot \frac{3n-1}{t_{0}^{n}}\cdot {\left(t-t_{0}\right)}^{n}]$$

It can be seen from Equation (8) that stress and the TOF of Lcr wave of the first item represents a linear function. The remainder items could be ignored, which are infinitesimal of higher order. In order for the convenience of discussion and analysis, a parameter ζ is defined in the following form:

(9) $$\zeta =\frac{t_{0}-t}{t_{0}}$$

where ζ expresses the TOF change rate of Lcr waves influenced by stress in a fixed acoustic path. The sum of Taylor series expansion in Equation (8) is:

(10) $$\sigma =\frac{1}{k}\cdot [\frac{2\zeta }{1-\zeta }+\frac{3\zeta^{2}}{{\left(1-\zeta \right)}^{2}}-\frac{3\zeta^{n+1}}{{\left(1-\zeta \right)}^{2}}-\frac{(3n-1)\zeta^{n+1}}{1-\zeta }]$$

In order to study the degree of the linear correlation between stress and Lcr wave TOF, the percentage of the contribution from the first item in Equation (8) needs to be determined. The formula can be written as:

(11) $$\eta =\frac{\frac{1}{k}\cdot 2\zeta }{\frac{1}{k}\cdot [\frac{2\zeta }{1-\zeta }+\frac{3\zeta^{2}}{{\left(1-\zeta \right)}^{2}}-\frac{3\zeta^{n+1}}{{\left(1-\zeta \right)}^{2}}-\frac{(3n-1)\zeta^{n+1}}{1-\zeta }]}\cdot 100\%$$

In actual measurements, when the Lcr wave propagates in a length of 120 mm in steel members, the TOF of Lcr wave in steel member under different stress and the corresponding parameter ζ are shown in Table 1. The percentage of the first few items (n) has been calculated by using Equation (11). The results are shown in Table 1.

It can be seen from Table 1 that the first item in Equation (8) plays a major role in the results. In addition, the effect of the second item gradually becomes larger with the increase of stress, but its influence is still negligible. According to the trend, the quadratic expression will be more accurate at larger stress values. In this study, structural steel member material is assumed to be in its elastic range. The remainder items have little influence on stress calculation and can be ignored. Therefore, Equation (8) can be further simplified to a linear equation:

(12) $$\sigma =\frac{2}{kt_{0}}(t_{0}-t)$$

Let $K=\frac{k}{2}$, and then K becomes the acoustoelastic constant for ultrasonic waves [18]. The acoustoelastic constant (K) functionally links the stress and TOF change, which should be measured by the uniaxial tensile test performed on the samples with the same materials as structural steel members. 

The relationship between stress and the TOF of an Lcr wave can be further simplified as:

(13) $$\sigma =B(t_{0}-t)$$

where B is defined as Stress to Acoustic Time Difference (SATD) factor, which represents the linear relationship between stress and the TOF of an Lcr wave:

(14) $$B=\frac{1}{Kt_{0}}$$

B is a combination of the acoustoelastic constant (K) and TOF (t_0_) in stress-free samples. In many studies [19,20,24], both the acoustoelastic constant (K) and elastic modulus (E) need to be calibrated by standard uniaxial tensile tests. In contrast, only the SATD factor (B) needs calibration in this study.

It can be seen from the above derivation that Equation (6) is a reduced form of Equation (1). By employing the fixed acoustic path method, Equation (6) becomes Equation (7). Equation (12) represents the linear term in the Taylor series expansion of Equation (7) about the point t=t_0_. Based on this, the linear relationship between stress and Lcr wave TOF is obtained in Equation (13). Therefore, Equation (13) is intrinsically a simplified representation of Equation (1). Compared with other theoretical formulae [19,20,35], the formula presented in this study has three main advantages. Firstly, the factors that influence stress measurement in Equation (1), including material density and the second and third order elastic constants of material, are integrated into a single SATD factor. This factor represents the linear relationship between stress and the TOF of an Lcr wave directly. Secondly, the calculation of SATD factors depends on experimental data fitting, instead of measurement of material density and the second and third order elastic constants of material. This simplifies the experimental work. Thirdly, Equation (13), the simplified representation of Equation (1), allows this method to be easily programmed and implemented.

## 3. Methodology

### 3.1. Development of Measurement System

**Figure 1:**
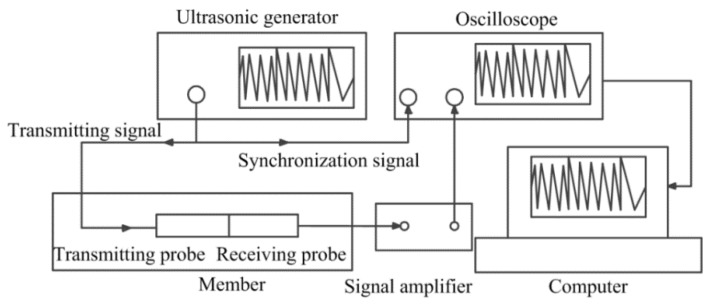
Measurement system schematic diagram.

**Figure 2:**
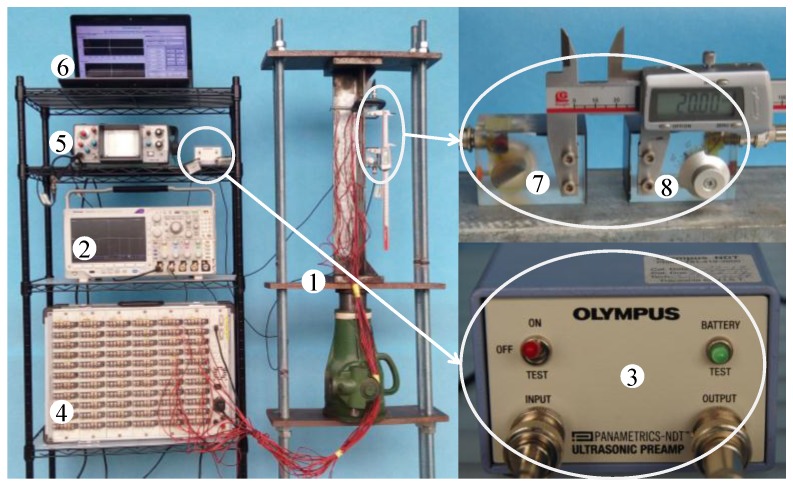
Measurement hardware system.

Based on above theory, a measurement system is designed and developed in this study. The measurement system includes both hardware and software systems. The schematic diagram and the photo of the developed hardware system are shown in Figure 1 and Figure 2, respectively. As can be seen, there are a total of eight components in hardware system. Specifically, (1) is the loading device; (2) is the oscilloscope (Tektronix company production, MDO3024); (3) is an ultrasonic preamplifier (OLYMPUS, 5670 PREAMP; New York, NY, USA); (4) is the static resistance strain gauge; (5) is the ultrasonic generator (Shantou Institute of Ultrasonic Instruments Co., Ltd., CTS-22; Shantou, China); (6) is a computer; (7) is the transmitting probe; and (8) is the receiving probe connected to the signal amplifier through wires. 

**Figure 3:**
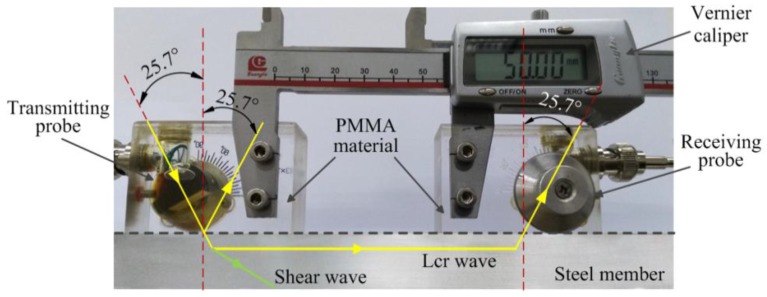
Lcr wave testing device.

The core device used to generate and receive Lcr waves is shown in Figure 3. Two ultrasonic transducers are attached to the test specimen. The role of the transmitting probe is to generate an ultrasonic wave at a specific frequency. The generated ultrasonic wave propagates from poly methyl methacrylate (PMMA) material to the steel, and then to the receiving probe via PMMA material. According to Snell’s law [34], the incident longitudinal wave angle is set as 25.7° so that Lcr wave can be generated and received. The nominal frequencies of the transducers for the probes are 5 MHz. Based on Javadi [37], the propagation depth of the Lcr wave generated in this study is about 1.1 mm beneath the member’s surface. The functions of the remaining components are summarized as follows: the loading device is used to generate measurable loading on the test specimens; the signal amplifier amplifies the received signals; the signal acquisition device can capture, display, and record signals from the transducers, by using the oscilloscope; and the computer is used to process the collected signal using the software system.

In this study, an in-house software is designed on the Labview platform, which consists of modules for signal generation, signal de-noising, pulse capturing, calibration of SATD factors, and stress calculation for in-service structural steel members. The signals generated can be of different forms, such as sine wave and square wave. In practice, a series of pulse signals are generated by the ultrasonic generator, and then propagate in the steel member. The propagating wave signals are captured at the receiving probe, and then transmitted to the computer. The received data are filtered by using wavelet transform method. The program is written in MATLAB software (MATLAB 7.14, The MathWorks, Natick, MA, USA) and embedded in Labview platform. This combination takes the advantages of both software, since MATLAB is more suitable for filtering while Labview is more suitable for data acquisition and instrument control.

To accurately measure the TOF of Lcr waves, the following process is proposed. In the beginning, the user controls the software system for signal generation. Then, the ultrasonic generator transmits a chain of pulse signals, and shunts them into a transmitting signal and synchronization signal by a transfer head. After wave mode conversion, the Lcr wave is generated. The Lcr wave, which propagates in a fixed acoustic path, is then received by the receiving probe. After being amplified, the received signal of the Lcr wave is displayed on the oscilloscope. In the above process, the synchronization signal of the incident wave is directly inputted to the oscilloscope. This results in two signals being displayed on the oscilloscope: one received signal and one synchronization signal. The time difference between these two signals is the TOF of the Lcr wave. The oscilloscope works with a resolution of 0.4 ns which allows very precise measurements of TOF.

### 3.2. Stress Measurement Method

**Figure 4:**
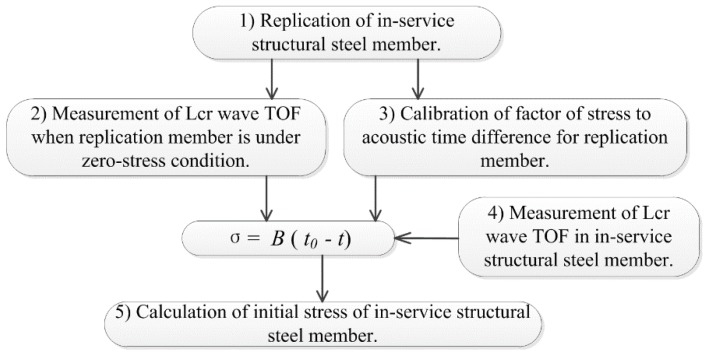
Flow chart of ultrasonic method.

Based on the theory and the developed measurement system, a five-step framework for the measurement of stresses of in-service structural steel members is proposed. The flowchart is shown in Figure 4. The detailed process is as follows:

(1) Replication of in-service structural steel member. Structural steel members are usually non-removable after installation. Since the calibration of SATD factors should ideally be performed on the original in-service member, a steel member with the same material and geometrical parameters to the in-service structural steel member should be used as a replication member.
(2) Measurement of Lcr wave TOF for the replication member under zero-stress condition, *i.e.*, t_0_ in Equation (13). Transmitting probe and receiving probe should be placed with a fixed distance apart on the replication member. Under stress-free conditions, the oscilloscope captures the arrival time of the transmitting wave signal and the synchronization signal. The acoustic time difference (ATD) between the two signals is t_0_.
(3) Calibration of the SATD factor for the replication member. This step is the core of the proposed method. The parameter to be calculated is B in Equation (13). A group of axial forces (σ_1_, σ_2_, …, σ_n_) are applied on the replication member that can be determined by strain gauges. Based on the tested axial forces and the corresponding Lcr wave TOFs obtained from step (2), a set of data ((t_1_, σ_1_), (t_2_, σ_2_),…, (t_n_, σ_n_)) can be obtained. The SATD factor (B) can then be easily calculated as the slope of the trend line of the data.
(4) Measurement of the Lcr waves TOF in the in-service structural steel member. The transmitting and receiving probes are placed on the in-service steel member with the same probe distance as that in step (2). The Lcr wave TOF in the in-service member (t in Equation (13)) can then be measured using the same procedure.
(5) Calculation of the internal stress of the in-service structural steel member. By substituting the obtained parameters: t_0_, B, and t, into Equation (13), internal stress of the in-service structural steel member can be determined.

## 4. Experimental Studies

### 4.1. Test Sample

In this study, five steel members and one aluminum plate are used as test specimens. To represent the actual conditions, the full-scale steel members are chosen as test specimens. Angle steel is widely used to build steel structure workshops, steel bridges, and electrical transmission towers in the building structure and engineering structure fields. It is used as not only the bearing member but also the connecting member, which makes it one of the most important and typical components. However, the current studies concerning internal stress measurement of angle steel are rare [12,18,19,20,21]. Therefore, this paper choose the angle steel member as one of the test specimens to study how to determine the internal stress using ultrasonic method. To investigate the factors that may affect the results, four steel plates and one aluminum plate are chosen as other test specimens. The dimensions of test specimens are determined by the bearing load value of the loading device and the probe size. The dimensions and material categories of the specimens are summarized in Table 2.

### 4.2. Validation of the Proposed Method

**Figure 5:**
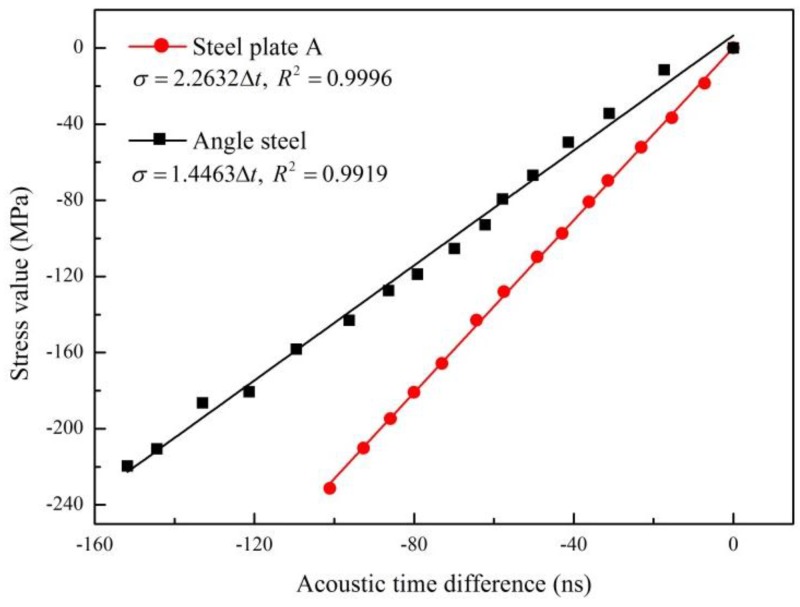
Fitting line between stress and TOF of steel plate A and angle steel.

To validate the proposed method, two structural steel members, steel plate A and angle steel, were firstly used to calibrate SATD factors. The distance between the transmitting and receiving probes was 100 mm for steel plate A and 120 mm for angle steel. The stress values and corresponding t_0_ of both specimens were measured, as shown in Figure 5. Using the least squares method, linear relationships between the stresses and t_0_ were fitted. Based on these relationships, the slopes can be obtained as the SATD factors, which are 2.2632 MPa/ns and 1.4463 MPa/ns for steel plate A and angle steel, respectively. Based on Equation (13) and the obtained SATD factor, the internal stress of in-service structural steel members can be easily calculated via the measurement of Lcr wave TOF (t), as shown in Table 3.

To verify the stress values measured by the proposed method, the standard strain gauge method was employed as a reference method. Several strain gauges were attached on the surface of both specimens, before load was applied to the steel member. The stresses of the steel members are calculated based on the product of Young’s modulus and the measured strain. It should be noted that the load applied to test specimens can produce uniformly distributed axial stress, so the surface and internal stresses are equal. 

The comparison results using both methods are listed in Table 3. The results show that the difference between the stresses measured by the two methods is less than 5% for every single test. This demonstrates the reliability and accuracy of the proposed method.

### 4.3. Investigation of Influencing Factors

To further investigate the factors that may affect the results, extensive experimental studies have been conducted in this section.

#### 4.3.1. The Positions of Probes

Structural steel members are generally slender with both ends fixed. The positions of the two probes on the in-service member and the replication member are not always identical. If the SATD factor is different in two positions, the stress values measured by ultrasonic method may be erroneous. Therefore, this subsection focuses on the influence of the probes’ positions on the SATD factor when the distance between the two probes is fixed.

**Figure 6:**
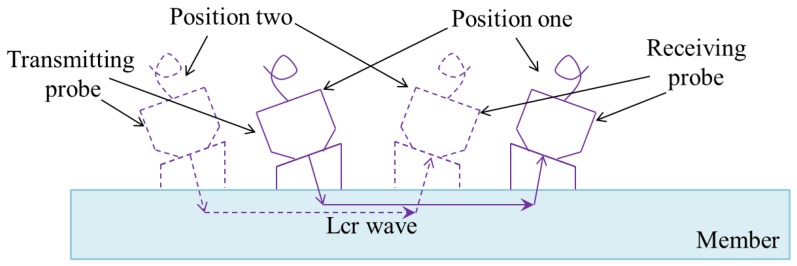
Position of probes.

**Figure 7:**
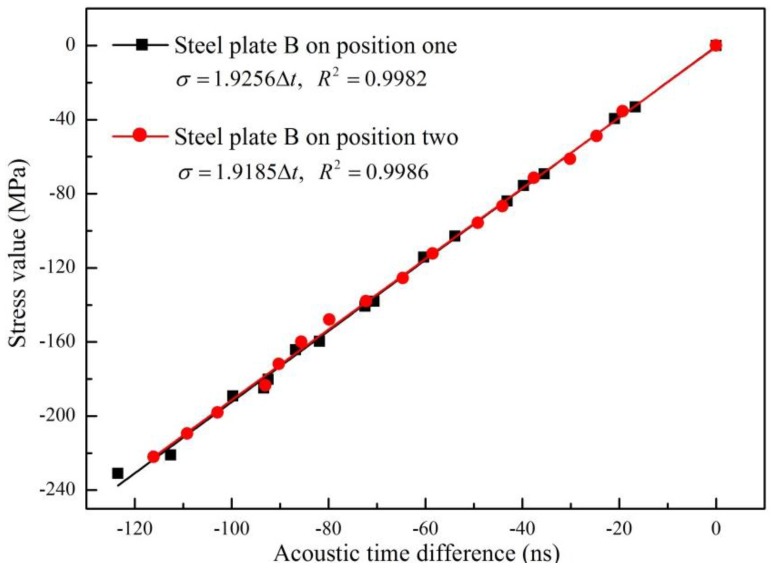
Fitting line between stress and TOF of steel plate B on position one and position two.

Steel plate C was used for this analysis. The distance between the transmitting probe and the receiving probe was set as 120 mm. Two different positions were marked on steel plate C. Position one was in the middle of steel plate C and position two was close to the edge, as illustrated in Figure 6. Under both conditions, the values of B and t_0_ and the corresponding stresses were obtained by using the proposed method, as shown in Figure 7. The fitted linear functions are almost identical, with a difference of only 0.512%. This small difference may be caused by the fitting process and can usually be ignored. 

Theoretically, stress is related to the SATD factor and t_0_ only, according to Equation (13). Both parameters are not sensitive to probe positions. This confirms that in actual stress measurement, the position of the probes does not affect the final result. To maximize the convenience of measurement, it is recommended to select probe positions that allow easy installation and removal.

#### 4.3.2. Materials

**Figure 8:**
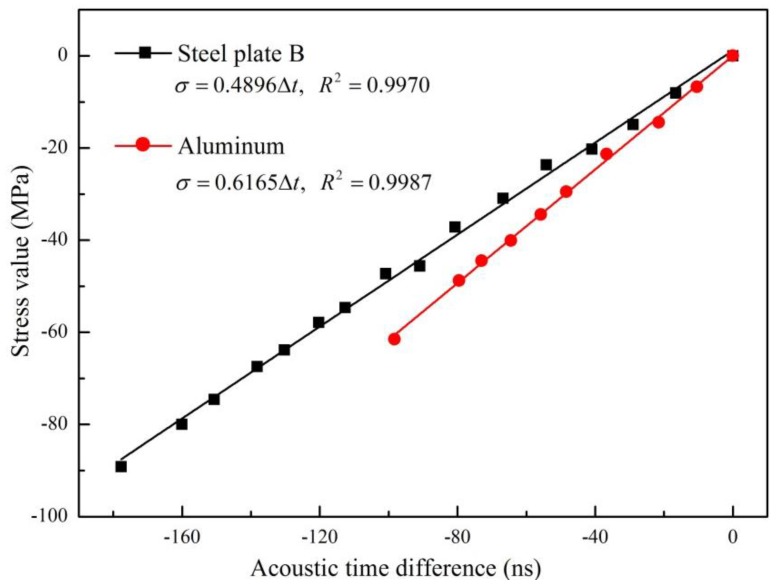
Fitting line between stress and TOF of steel plate B and aluminum plate.

Equation (14) shows that the SATD factor is influenced by the second and third order elastic constants of materials. To investigate its influence, the SATD factors were calibrated for steel plate B and the aluminum plate. The dimensions of plate steel B and the aluminum plate are the same, as shown in Table 2. The distance between the two probes was set as 120 mm. The results obtained using the proposed method and the fitted linear relationships are shown in Figure 8.

As can be seen, the SATD factor of steel plate B and the aluminum plate are 0.4896 MPa/ns and 0.6165 MPa/ns, respectively. The difference between them is 22.801%. This means that material properties have a direct influence to the SATD factor. This matches with the conclusion from the theoretical study. Furthermore, a previous study [26] suggests that ultrasonic wave propagation characteristics will vary depending on the steel’s carbon content. Therefore, the SATD factor should be calibrated independently for each structural steel member.

In the welding field, welding procedure has a severe effect on the parent material. The acoustoelastic constant of the weld zone differs considerably from the parent material and the heat affected zone as well. This practical problem in ultrasonic stress measurement limits it to laboratory applications only. However, this problem could be minimized in the measurement of steel structural members’ internal stresses. Standardized production of structural steel members makes the material of each member almost the same. So it is not necessary to measure every acoustoelastic constant or SATD factor, which enables the proposed method to be applied in engineering practices.

All the results shown in Figure 5, Figure 7 and Figure 8 exhibit a nearly perfect linear relationship between the stress and TOF of propagating Lcr wave, with less than a 1% fitting error. This is consistent with other scholars’ research results [19,20,21,22] and theory of acoustoelasticity, which demonstrate that the proposed method is suitable for the stress evaluation of steel members with different shapes, materials, and probe positions.

#### 4.3.3. Distance between Probes

**Figure 9:**
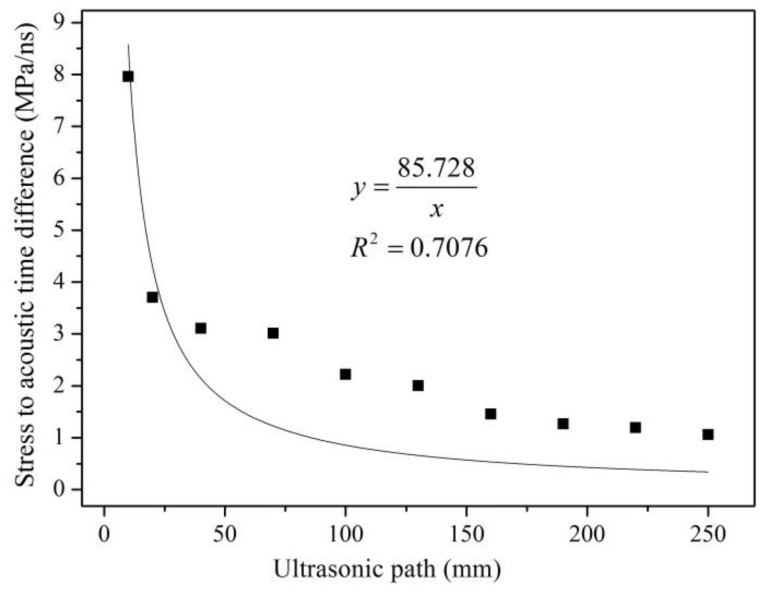
Fitting trend line between probe distance and the SATD factor of steel plate C.

**Figure 10:**
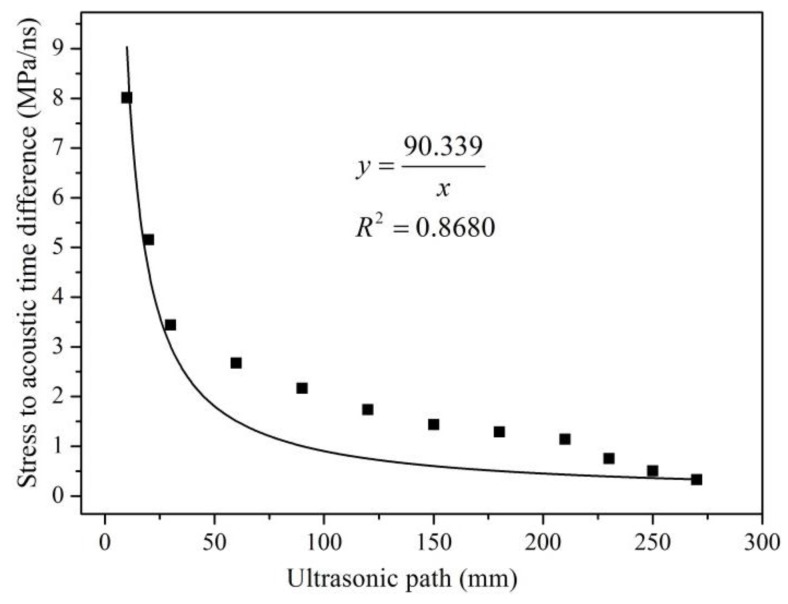
Fitting trend line between probe distance and the SATD factor of steel plate D.

Steel plate C and steel plate D were employed in this study to investigate the influence of different probe distances on the accuracy of the proposed method. The SATD factors and their corresponding distances between the two probes was measured using the proposed framework. The obtained results are shown in Figure 9 and Figure 10.

By substituting $t_{0}=\frac{L}{V_{0}}$ into Equation (14), a new form of the SATD factor can be obtained:

(15) $$B=\frac{V_{0}}{KL}$$

Equation (15) shows that when ultrasonic waves propagate in a single medium, ultrasonic wave velocity V_0_ and the acoustoelastic constant (K) are constants. The SATD factor decreases as the distance between the two probes increases in the form of an inversely proportional function. Figure 9 and Figure 10 illustrate that the SATD factor decreases as the ultrasonic path length increases. However, the figures also show obvious discrepancies between the fitted functions and the experimental results, which confirms the complexity of the problem. The discrepancies may come from inaccurate consideration of the ultrasonic path. As shown in Figure 3, the ultrasonic path consists of three parts: propagation distance in the air, PMMA, and the steel member. Ultrasonic velocity in the air and PMMA is unchanged, while the steel member changed with stress. Therefore, the accurate ultrasonic path should not include propagation distance in air and PMMA. However, this is not easy for implementation in practice. In order to accurately evaluate the relationship between the SATD factor and the ultrasonic path length, the calculation and calibration of the effects from air and PMMA should be investigated in more detail in future studies.

It should be noted that the inclusion of wave propagation in air and PMMA does not affect the results in previous cases. The reason is that their ultrasonic path lengths are the same in each case. So the effects are self-eliminated.

### 4.4. Discussions

There are some advantages of the proposed ultrasonic method, compared with other methods, to measure internal stress of in-service structural steel members. Firstly, it is a non-destructive method. The internal stress of steel members, measured by the proposed ultrasonic method, was compared with those obtained by the standard strain gauge method. The results from the two methods show good agreement. As the Lcr wave is less sensitive to texture and most sensitive to stress, the proposed method offers advantages not shared with other ultrasonic based techniques, such as shear wave method [12,20]. Secondly, the test procedure is straightforward, without the measurement of second and third order elastic constants. Thirdly, the measurement system consists of five portable devices, which is convenient for field measurement. Finally, the cost of the developed equipment is relatively low, compared with X-ray diffraction and magnetic-elastic method. Although it is a promising method which may find immediate industry application, the implementation of the proposed method needs careful calibration. The reason is that the propagation characteristics of ultrasonic wave modes vary in different materials and/or in different shapes [38,39,40]. The extension to more general models requires a more detailed investigation of wave propagation, specifically when it applies to higher gradient and micromorphic materials, as discussed in [41,42]. 

It should be pointed out that calibration of structural steel members is more accurate than that in the welding field. Material properties near the welding zone change in different degrees, which are difficult to simulate. The zero-stress reference points of changed material properties are often unknown. Thus, the fitting parameters may not reflect the true condition of the material, which leads to inaccurate measurement results. In contrast, the above problems are not dominant in internal stress measurement. Steel members are standardized; their material properties are constant; and thus the zero-stress reference point can be obtained. The simplified relationship between stress and the TOF of an Lcr wave, *i.e.*, Equation (13), makes the calibration of SATD factor convinient.

## 5. Conclusions

This paper presents a non-destructive evaluation method to determine the internal stress of in-service structural steel members using Lcr waves. Based on the theory of acoustoelasticity, a measurement system is designed and developed. Using the developed equipment and the proposed methodology, tests of ultrasonic stress measurement were performed on six specimens with different geometries and materials, as well as varying probe positions and distances. Experimental results show that the TOF of Lcr wave and its corresponding stress in steel member exhibits an almost perfect linear relationship with less than a 1% fitting error. The strain gauge method was employed to validate the results measured by the ultrasonic method. The obtained results show that the difference is within 5%, which confirms the effectiveness of the proposed method. The experimental results from this study show that the proposed measurement process is convenient and quick, and that the results are reliable. Parametric studies are performed to investigate the factors that may influence testing results. Experimental results show that the position of the probes on the members will not affect results when the distance between the probes is fixed. On the other hand, the distance has an inversely proportional relationship with the SATD factor. In addition, material properties affect the stress measurement results through the SATD factor. The experimental results are consistent with the theory of acoustoelasticity.

Since in-service structural steel components are not removable and the determination of internal stress is critical, the proposed method will find immediate application in many construction and industrial areas. Future research efforts should focus on the measurement of two-dimensional and three-dimensional stresses using ultrasonic methods, when structural steel members are subjected to more complex loading conditions. The relationships between the test results and other influencing factors including temperature and coupling medium, are also recommended for future study.

## Figures and Tables

**Table 1:** The contribution percentage of the first item in Equation (8) under different stresses.

σ (MPa)	t (ns)	ζ	η			
			(*n* = 1)	(*n* = 2)	(*n* = 10)	(*n* = 100)
0.00	40,534	0.0000	-	-	-	-
−11.54	40,552	−0.0004	100.00%	100.10%	100.10%	100.10%
−49.58	40,576	−0.0010	100.00%	100.25%	100.25%	100.25%
−105.40	40,604	−0.0017	100.00%	100.43%	100.43%	100.43%
−158.29	40,635	−0.0025	100.00%	100.63%	100.63%	100.63%
−219.64	40,686	−0.0037	100.00%	100.93%	100.93%	100.93%

**Table 2:** Test samples material and dimension.

Name	Material	Dimension
Steel plate A	Q235 steel	400 mm × 40 mm × 8 mm
Angle steel	Q235 steel	∠80 mm × 80 mm × 6 mm
Steel plate B	Q235 steel	450 mm × 40 mm × 12 mm
Steel plate C	Q235 steel	600 mm × 40 mm × 12 mm
Steel plate D	Q235 steel	600 mm × 40 mm × 20 mm
Aluminum plate	6061	450 mm × 40 mm × 12 mm

**Table 3:** Comparison between two methods of steel plate A and angle steel.

Ultrasonic Method (Steel Plate A) (MPa)	Strain Gauge Method (Steel Plate A) (MPa)	Difference (Steel Plate A) (%)	Ultrasonic Method (Angle Steel) (MPa)	Strain Gauge Method (Angle Steel) (MPa)	Difference (Angle Steel) (%)
−81.08	−78.93	2.72	−67.28	−69.13	2.68
−107.20	−108.64	1.32	−95.05	−95.16	0.11
−143.24	−142.41	0.59	−103.73	−107.84	3.81
−186.48	−182.01	2.46	−115.88	−121.16	4.36
−189.18	−188.86	0.17	−132.65	−129.91	2.11
−197.29	−189.87	3.91	−156.37	−160.63	2.65
−209.91	−210.67	0.36	−178.36	−182.92	2.49
−227.92	−232.28	1.88	−194.56	−188.98	2.95
-	-	-	−211.91	−212.81	0.42
-	-	-	−214.80	−221.60	3.07

## Abbreviations

The following abbreviations are used in this manuscript:

NDT:	non-destructive testing
Lcr waves:	Longitudinal critically refracted waves
TOF:	time-of-flight
SATD factor:	Stress to Acoustic Time Difference factor
PMMA:	poly methyl methacrylate
ATD:	acoustic time difference
